# Information Needs and Concerns of Patients with Inflammatory Bowel Disease: What Can We Learn from Participants in a Bilingual Clinical Cohort?

**DOI:** 10.1371/journal.pone.0150620

**Published:** 2016-03-03

**Authors:** Valérie Pittet, Carla Vaucher, Michel H. Maillard, Marc Girardin, Philippe de Saussure, Bernard Burnand, Gerhard Rogler, Pierre Michetti

**Affiliations:** 1 Institute of Social & Preventive Medicine (IUMSP), Lausanne University Hospital, Lausanne, Switzerland; 2 Division of Gastroenterology & Hepatology, Lausanne University Hospital, Lausanne, Switzerland; 3 Division of Gastroenterology & Hepatology, Geneva University Hospital, Geneva, Switzerland; 4 Division of Gastroenterology & Hepatology, University Hospital Zurich, University of Zurich, Zurich, Switzerland; 5 Crohn and Colitis Center, Clinique La Source-Beaulieu, Lausanne, Switzerland; University Hospital Llandough, UNITED KINGDOM

## Abstract

**Background:**

Inflammatory Bowel Disease (IBD) patients are confronted with needs and concerns related to their disease.

**Aim:**

To explore information expectations of patients included in a national bilingual IBD cohort in Switzerland (SIBDC).

**Methods:**

This is a mixed-methods study, comprising 1) a semi-narrative survey sent to 1506 patients from the SIBDC and 2) two focus groups conducted with 14 patients to explore and assess the relevance of the survey’s findings. Data collected within the framework of the SIBDC was used to characterize survey’s responders.

**Results:**

728 patients (48%) replied to the survey: 52.5% females, 56% Crohn’s disease (CD), 87% secondary/tertiary level educated, 70% full/part-time employed. On average, 47% of patients sought for information, regardless of the disease stage; 27% of them were dissatisfied with information received at the time of first symptoms. During flares, 43% were concerned about drugs and therapies; in remission, 57% had concerns on research and developments; 27% searched for information linked to daily disease management. Information-seeking increased when active disease, for CD with high levels of perceived stress (OR = 2.47; p = 0.003), and for all with higher posttraumatic stress symptoms. The focus groups confirmed a perceived lack of information about general functioning, disease course, treatments and their risks, extra-intestinal symptoms and manifestations.

**Conclusions:**

Information remains insufficient for IBD patients. Lack of information in specific domains can potentially cause stress and hinder detection of symptoms. Better information should be considered as a potentially important component in improving patients’ outcomes in IBD.

## Introduction

Patients suffering from Inflammatory Bowel Disease (IBD), including both Crohn’s Disease (CD) and Ulcerative Colitis (UC), are confronted with numerous worries and concerns related to the origins of their disease, its chronic nature and long-term progression. In addition to that, IBD patients may have more specific information needs related to medications and daily disease management. Studies exploring information needs, level of knowledge, or sources of information in IBD patients started 40 years ago [1,2], but only a few were conducted more recently. These studies were mostly focused on a limited number of patients, a single center or region, or used a pre-selected list of items to assess patients’ needs. They showed that about 75% of the patients would have welcomed more information on their disease [1–5], including on the risks and benefits of the various treatments [6,7], disease aetiology, symptoms and possible diets [3,8] or self management [9]. IBD patients have many concerns related to their chronic disease such as loss of bowel control, having to wear an ostomy bag, reduced energy levels[10], body image, isolation and fear, not reaching full potential, feeling dirty or having lack of information from the medical community [11]. There also appears to be differences in information needs between UC and CD patients [8]. CD patients seemed to search for more information about heredity, long-term disease progression, risks of cancer and other complications [12] whereas UC patients seemed more interested in medications [13].

Gastroenterologists were patients’ favourite source of information [3,9,14,15], although patients were also opened to other sources such as books, internet or nurses. Having sufficient or adequate information was shown to potentially increase compliance to treatments [16]. In other chronic diseases like cancers, searching for health-related information was associated with being more health-conscious and more compliant [17–19]. Other studies showed that having access to health-related information on IBD negatively impacted on quality of life or anxiety levels [20–22]. Indeed, more information might affect illness perceptions, which were found to directly influence depression and anxiety levels as well as family functioning [23]. Health-related information might have positive and negative effects, therefore characteristics of those patients who searched for information and types of information searched need to be further explored, especially nowadays with the abundance of new information and communication technologies to which patients are daily confronted.

The aim of our study was to take the opportunity of conducting a national IBD cohort study 1) to explore and assess information needs and concerns, sources and satisfaction in a large number of patients, using an approach combining a narrative survey and patients focus groups, to circumvent the limitations of prior studies, and 2) to characterize patients who searched for this information.

## Methods

### Study design and population

This is a mixed-methods study combining quantitative results from a cross-sectional semi-narrative survey conducted among patients included in the Swiss Inflammatory Bowel Diseases Cohort (SIBDC) Study [24], complemented by the qualitative results from two focus groups (FG) discussions with IBD patients. Patients enrolled in the SIBDC were males and females, with a diagnosis established for at least 4 months prior to enrolment and confirmed radiologically and/or endoscopically and/or surgically. Enrolment was conducted by treating gastroenterologists, in private practices, regional or university hospitals.

### Semi-narrative survey

The survey was conducted in October 2009 on all 1506 adult patients included in the SIBDC at that time. It was developed based on the methods of critical incident reporting [25,26] and contained questions on sources and topics of information sought for by patients in four critical disease stages: 1) the time of first symptoms, 2) at diagnosis, 3) during an active disease phase and 4) in remission. Topics of information searched were collected as follows: patients were first asked to remember if they had sought information (yes, no, do not remember), and were then asked to describe thoroughly the information sought. Content analysis was performed by extracting keywords out of open-ended questions and by grouping them, first in sub-topics and second in main topics. A double check of the classifications in sub- and main themes was performed by comparing them with those independently assessed by a psychologist having previous experience in qualitative research. The number of sub-topics covered by the survey was 43, which were grouped into 6 main information categories: 1) research and development on IBD, 2) therapies, 3) tips for daily disease management, 4) sharing of experience, 5) basic information on the disease, 6) miscellaneous. Each patient could have sought single or multiple topics of information. We created 6 binary variables to calculate the proportions of each main topic consulted. Information sources were collected using the following categories: “Family doctor”, “Gastroenterologist” (“physicians”), “Internet”, “Pharmacist”, “Books”, “TV” (“books & TV”), “Patients associations”. Linkage with SIBDC data was made by using the unique ID number assigned to all patients at enrolment in the cohort.

### Focus Groups

We used FG to explore and check patients’ overall needs and concerns related to their disease. FG management recommendations [27,28] stated that FG should comprise 6 to 12 participants. Therefore we performed batch contacts to reach the expected number of participants for each disease FG. A total of 71 patients were contacted by postal mail: 10 answered and 8 finally took part in the UC group; 8 answered and finally 6 took part in the CD group discussion. Inclusion criteria were having been followed regularly by a gastroenterologist for the past 18 months, having been diagnosed at least 5 years ago, and having experienced at least 2 different categories of treatments. The two FG were conducted in a neutral context and discussions were framed by the study investigator (VP) and a moderator (CV), experienced in qualitative research. FG took place between May and June 2014 and lasted approximately 2 hours each. FG discussions were audio-recorded and fully transcribed with the participants’ written consent. Content analyses were performed. Emerging themes were drawn and thematic categories were created to cover the main topics being addressed. Each FG was analyzed individually and interactions between participants were observed. Categories of interest and opinions were then compared between UC and CD patients.

### Cohort data

Data on patients and disease characteristics were extracted from the SIBDC database. These data had been previously collected through gastroenterologists or trained study nurses during the enrolment medical visit. These comprised disease location and phenotype for CD, disease extension for UC, presence of extra-intestinal manifestations and disease duration. From the self-administered questionnaire completed by all patients at enrolment in SIBDC, we used the following data: age, gender, language (French / German speakers, referring to the language of questionnaire completion), age at diagnosis, family history of IBD, smoking status, marital status (married / not married), education level (None or compulsory / upper secondary education level / tertiary education), working status (Employed / In training / at home or unemployed / retired). Both education and working status classifications were derived from those commonly used in the Swiss national statistics.

The self-administered enrolment questionnaire also included a large set of validated mental health questionnaires, some of which were used for the current study. The Hospital Anxiety and Depression Scale (HADS) was used to assess for anxiety and depression. This questionnaire contains 14 items scaled 0 to 3, 7 for each score, grouped into 3 categories (0–7: normal, 8–10: mild, 11–21 moderate to severe anxiety or depression symptoms) [29]. Perceived stress was assessed using the 30-item Perceived Stress Questionnaire (PSQ) [30]. PSQ mean score was categorized as follows: 0–0.3: None to low stress; 0.3–0.4: Low to average stress; 0.4–0.51: Average to high stress; >0.51: High stress. Global social support was assessed using the 7-item ENRICHD Social Support Inventory (ESSI) [31], and the ESSI score was treated as continuous. General quality of life was assessed using the SF-36 questionnaire, and scores were summarized and normalized into a Physical Component Score (PCS) and a Mental Component Score (MCS). Disease-related quality of life was assessed using the 32-items IBDQ questionnaire [32] grouped into 4 sub-scores (bowel-related symptoms, systemic symptoms, emotional function, and social function). Finally, clusters of post-traumatic stress symptoms were assessed using the Post-Traumatic Stress Diagnostic Scale (PDS). This questionnaire contains 17 questions rated on a 4-point Likert Scale, which were grouped into three continuous sub-scores: re-experiencing, avoidance, and hyperarousal scores [33].

### Statistical analysis

Descriptive analyses including tabulations with numbers and percentages were performed to characterize the study population. Characteristics of respondents versus non-respondents to the questionnaire were compared for all factors considered in the present study. Satisfaction with information and sources of information was compared according to gender and diagnostic. Differences in proportions were calculated using a z-test for testing the null hypothesis of no difference. A p-value <0.05 was considered statistically significant. Multivariate analyses were performed using logistic regression modelling to examine the strength of the association between information-seeking at the different stages and all independent exposure factors. Odds ratios (OR) and 95% confidence intervals (CI) for information-seeking, adjusted for gender, age and disease duration were calculated. Analyses were conducted using STATA statistical software v.13.1 (STATA Corp. Texas, USA).

### Ethics approval

Ethics approval was obtained from the regional Swiss Ethics Committees in which cohort participants were enrolled. Ethics approval was obtained to conduct focus groups with patients of the cohort leaving in the Canton of Vaud (Protocol n°185/13).

## Results

### Characteristics of the sample population

A total of 728 / 1506 patients (48%) responded to the survey, 407 (55.9%) with CD and 321 (44.1%) with UC (Table 1).

**Table 1:** Comparison of patients’ characteristics between responders and non-responders to the questionnaire on information needs. Values are number and percentages unless specified.

Variables	All	Responders	Non-responders	p-value
**All**	1506	728 (48.3)	778 (51.7)	
**German speakers**	954 (63.3)	460 (63.2)	494 (63.5)	0.618
**Female gender**	763 (50.7)	382 (52.5)	381 (49.0)	0.174
**Age**				<0.001
*<35 years*	532 (35.3)	217 (29.8)	315 (40.5)	
*35–50 years*	559 (37.1)	292 (40.1)	267 (34.3)	
*>50 years*	415 (27.6)	219 (30.1)	196 (25.2)	
**Age at diagnosis**				0.029
*< = 40 years*	1198 (79.6)	562 (77.2)	636 (81.8)	
*>40 years*	308 (20.4)	166 (22.8)	142 (18.2)	
**Disease duration**				0.231
*<5 years*	511 (33.9)	234 (32.1)	277 (35.6)	
*5–15 years*	581 (38.6)	281 (38.6)	300 (38.6)	
*>15 years*	414 (27.5)	201 (25.8)	201 (25.8)	
**Diagnostic**				0.076
*CD*	877 (58.2)	407 (55.9)	470 (60.4)	
*UC*	629 (41.8)	321 (44.1)	308 (39.6)	
**Extra-intestinal manifestations**	698 (46.4)	335 (46.0)	363 (46.7)	0.803
**Family history of IBD**	190 (13.7)	85 (12.7)	105 (14.6)	0.309
**Married**	600 (50.4)	366 (53.2)	234 (46.6)	0.025
**Education level**				0.005
*None or compulsory*	181 (14.8)	85 (12.0)	96 (18.6)	
*Upper 2nd education*	674 (55.2)	399 (56.5)	275 (53.4)	
*Tertiary education*	366 (30.0)	222 (31.4)	144 (28.0)	
**Working status**				0.914
*Employed*	840 (69.8)	485 (70.0)	355 (69.5)	
*In training*	62 (5.2)	35 (5.0)	27 (5.3)	
*At home/unemployed*	157 (13.0)	87 (12.6)	70 (13.7)	
*Retired*	145 (12.0)	86 (12.4)	59 (11.5)	

About two-thirds of the responders were German-speakers; half were women, and 30% were less than 35 years old. Almost three quarters (N = 562) of the patients were diagnosed before 40 years old, slightly less than non-responders (p = 0.029), and one quarter (N = 201) had a disease duration > 15 years. About 42% of CD patients had a penetrating disease (N = 169) and 54% had a disease involving the ileum. Thirty-eight percent of UC patients (N = 121) had an extensive colitis. Seventy percent of the patients were fully or partially employed, and 85% were secondary/tertiary level educated. Among those who responded to the survey, 346 (48%) searched for information at the time of first symptoms and 385 (53%) at the time of diagnosis. Referring to their current or more recent situation, 382 (52%) documented that they searched for information when the disease was active and 284 (39%) when the disease was in remission. Overall, 49% of the patients who searched for information at the time of first symptoms were fully satisfied with it, 24% partially satisfied and 27% not satisfied at all. Men were more frequently fully satisfied than women (57% versus 42%; p = 0.007), as were UC patients (55% versus 43% for CD; p = 0.044). Main reasons for dissatisfaction were related to diagnostic delay (e.g., due to misdiagnosis or lack of consideration of symptoms by the family doctor or specialists), lack of knowledge on the disease among physicians and in the general population at that the time, and unclear information available through the internet.

### Sources of information

The most frequent sources of information consulted by the patients at the time of first symptoms were physicians (66%), internet (37%) and books or television (30%), Table 2.

**Table 2:** Comparison of sources of information consulted by the 346 patients who searched for information at the time of 1^st^ symptoms, and the 385 patients who searched for information at the time of diagnostic.

	All	Gender			Diagnosis		
Sources of information	N (%)	Female N (%)	Male N (%)	p-value	CD N (%)	UC N (%)	p-value
**TIME OF 1**^**ST**^ **SYMPTOMS**							
*Physicians*	228 (65.9)	125 (65.4)	103 (66.4)	0.844	125 (67.6)	103 (64.0)	0.482
*Paramedics*	18 (5.2)	13 (6.8)	5 (3.2)	0.152	7 (3.8)	11 (6.8)	0.231
*Family / friends / Patients association*	26 (7.5)	15 (7.8)	11 (7.1)	0.840	16 (8.6)	10 (6.2)	0.421
*Internet*	127 (36.7)	54 (28.3)	73 (47.1)	**<0.001**	57 (30.8)	70 (43.5)	**0.015**
*Books / TV*	103 (29.8)	62 (32.5)	41 (26.4)	0.224	59 (31.9)	44 (27.3)	0.355
**Full satisfaction with**							
*Physicians*		52 (45.2)	58 (60.4)	**0.027**	53 (46.4)	57 (58.7)	0.075
*Paramedics*		1 (41.7)	5 (20.0)	0.394	3 (42.8)	3 (30.0)	0.585
*Family / friends / Patients association*		6 (46.1)	4 (50.0)	0.864	5 (45.4)	5 (50.0)	0.835
*Internet*		19 (39.6)	43 (62.3)	**0.015**	27 (50.0)	35 (55.6)	0.548
*Books / TV*		22 (38.6)	22 (61.1)	**0.034**	22 (40.7)	22 (56.4)	0.135
**AT DIAGNOSIS**							
*Physicians*	123 (31.9)	71 (31.3)	52 (32.9)	0.735	80 (37.2)	43 (25.3)	**0.013**
*Paramedics*	29 (7.5)	17 (7.5)	12 (7.6)	0.969	12 (5.6)	17 (10.0)	0.103
*Family / friends / Patients association*	57 (14.8)	36 (15.9)	21 (13.3)	0.485	33 (15.4)	24 (14.1)	0.736
*Internet*	175 (45.4)	93 (41.0)	82 (51.9)	**0.034**	90 (41.9)	85 (50.0)	0.111
*Books / TV*	92 (23.9)	58 (21.5)	34 (25.6)	0.362	51 (23.7)	41 (24.1)	0.928

Internet was more often consulted by men (47% versus 28%; p<0.001) and by UC patients UC (43% versus 31%; p = 0.015). Women were not as satisfied as men with information they received from physicians (45% versus 60%; p = 0.027), internet (40% versus 62%; p = 0.015) and books or TV (39% versus 61%; p = 0.034). At the time of diagnosis, the most frequent sources of information were internet (45%), physicians (32%) and books or TV (24%). Family, friends and other patients were more often consulted at that time, as compared to the time of first symptoms (15% versus 7%; p = 0.002). At diagnosis, CD patients more often consulted physicians (37% versus 25%; p = 0.013) than UC patients and men more often consulted the internet, as compared to women (52% versus 41%; p = 0.034). A minority of participants consulted patients associations or pharmacists.

### Topics of information

**Fig 1:**
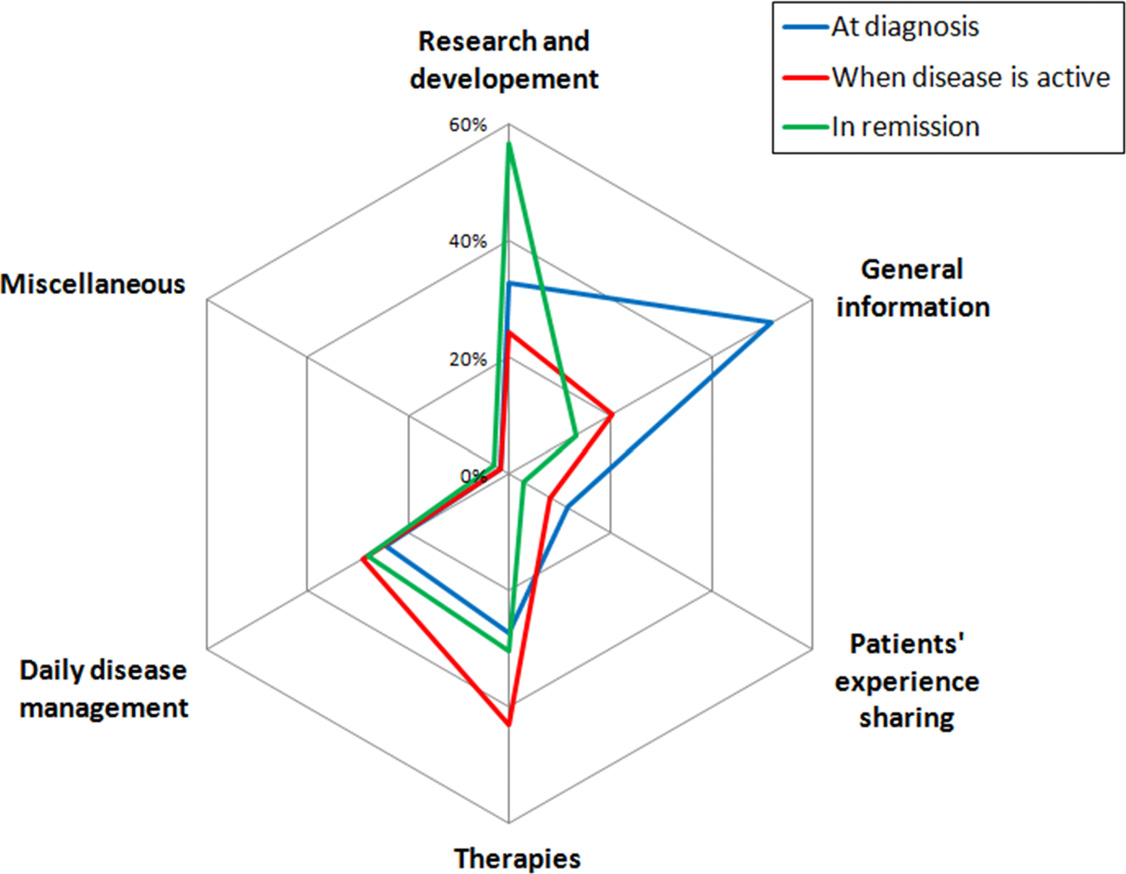
Topics of information searched according to disease phases.

Patients were more likely to search for general information about the disease at the time of diagnosis, whereas they sought for information about drugs more frequently during a flare. Finally, they appeared to be more frequently interested in information about research and development during periods of remission (Fig 1).

Main frequent sub-topics searched by patients at the time of diagnosis, Table 3, were linked to general aspects of the disease (50% of all searches at that time), drugs or other therapies (23%) and long-term disease progression (22%). During the active disease phase, searches focused on drugs or other therapies (27%), general aspects of the disease (18%), and risk factors and prevention of flares (14%). When in remission, patients were mostly interested in clinical trials / research on new drugs (46%), recent advances in research (25%) and long-term disease evolution (20%).

**Table 3:** Topics of information consulted by the patients who searched for information, according to phase of the disease.

	At the time of diagnosis	When disease is active	When disease is quiet
**Total number of patients who sought for information**	385	382	284
**Research and development on IBD**	126 (32.7)	93 (24.3)	161 (56.7)
Long term disease progression	85	41	57
Origins of the disease	35	17	12
Epidemiological/statistical results	14	4	2
Can we cure the disease?	11	12	15
Trends in mortality	10	4	0
Recent advances in research	6	11	71
Hereditary	6	4	5
Clinical trials / research on new drugs	5	23	74
**Therapies**	105 (27.3)	164 (42.9)	86 (30.3)
Adverse events of drugs	15	39	15
Information on drugs or other therapies	87	104	53
Information on surgery	12	40	9
Alternative or complementary medicines	15	48	24
Information on change / stop of treatment	0	4	14
**Tips for daily disease management**	95 (24.9)	111 (29.1)	80 (28.2)
Nutrition	45	37	30
Risk factors and prevention of flares	20	52	40
Daily management	19	25	19
Quality of life and consequences on life habits	18	20	18
Gynaecology / Pregnancy	12	10	5
Psychological factors / psychological help	11	21	24
Communication aspects (in the family, at work)	1	0	1
Legal aspects (absence at work, disability)	1	5	5
**Basic information on the disease**	200 (52.0)	78 (20.4)	37 (13.0)
Generalities about the disease	193	64	29
Extra-intestinal manifestations	10	10	3
Cancer	4	7	3
Vaccination	1	0	3
**Patients experience sharing**	44 (11.4)	31 (8.1)	8 (2.8)
Testimonies	26	13	1
Exchanges	17	16	7
Support groups	5	5	1
**Miscellaneous**	7 (1.8)	7 (1.8)	9 (3.2)

**Fig 2:**
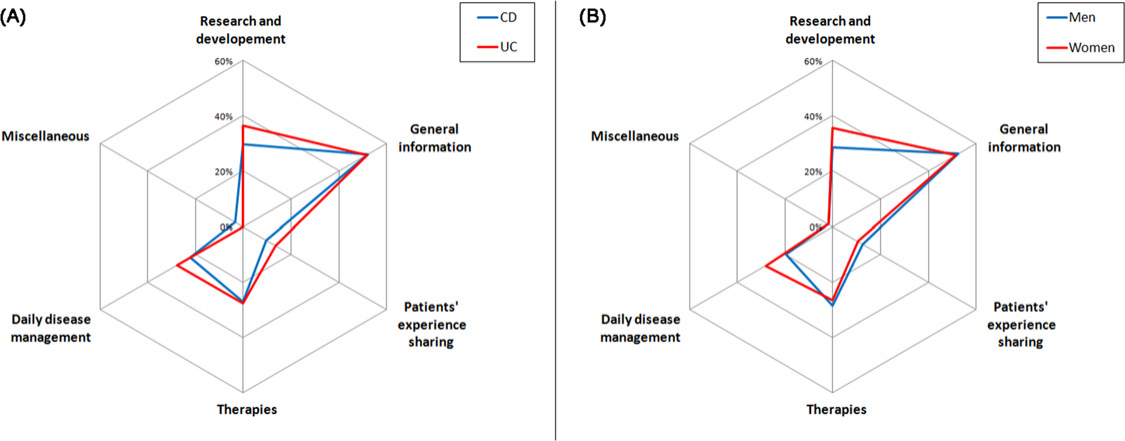
Topics of information searched at diagnosis, according to gender (A) and type of disease (B).

**Fig 3:**
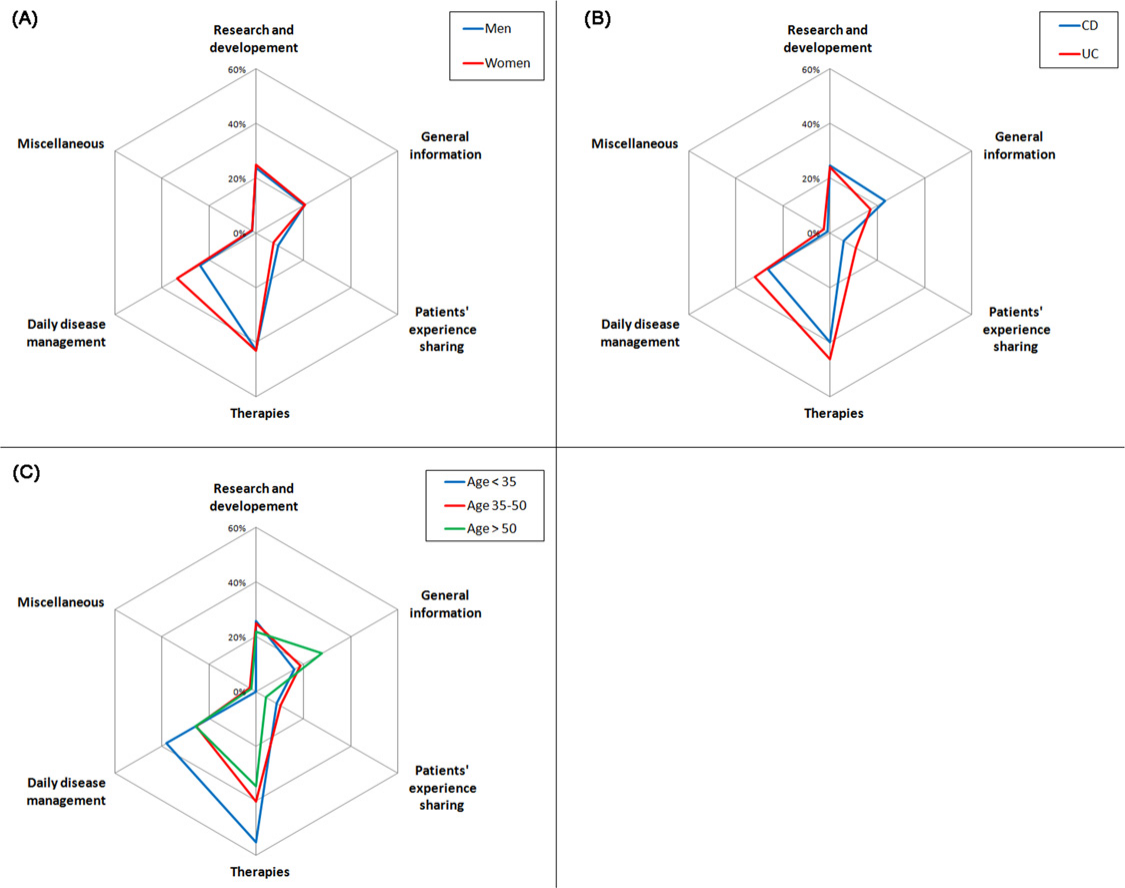
Topics of information searched during active disease phases by gender (A), type of disease (B) and age (C).

**Fig 4:**
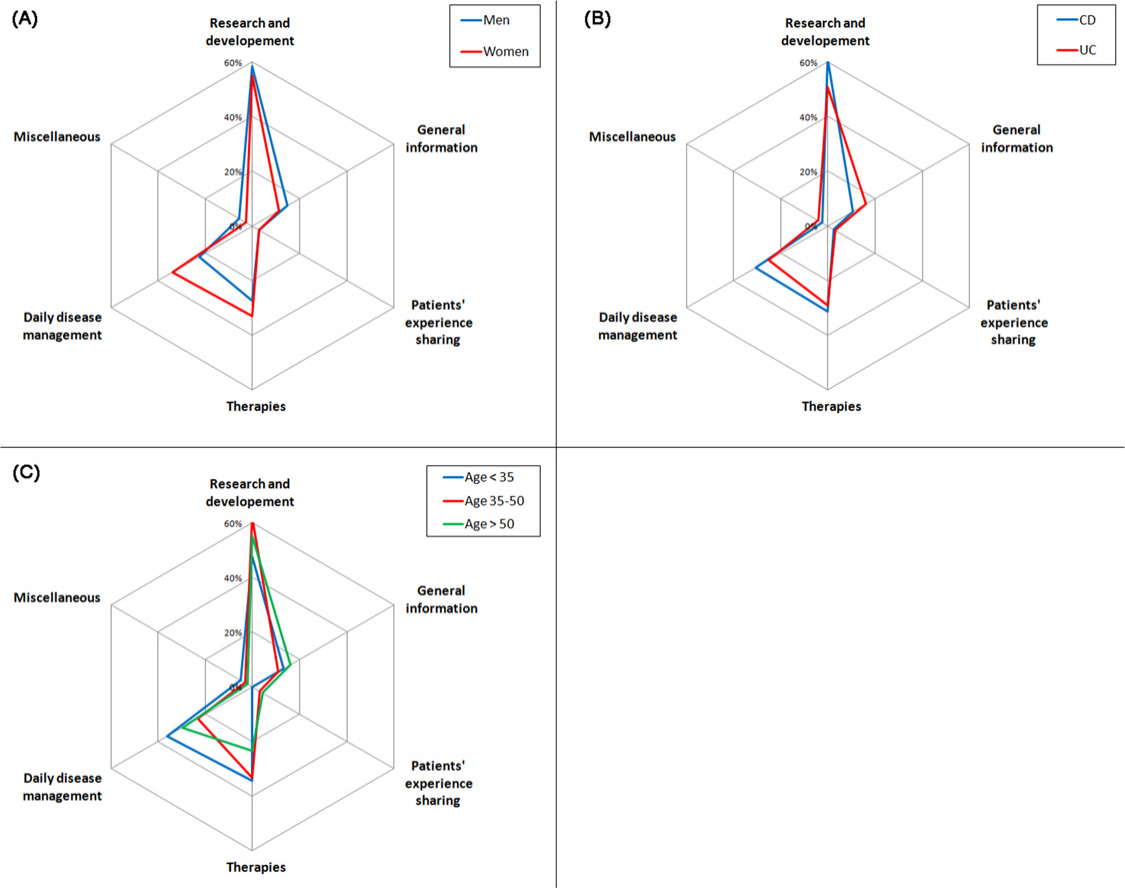
Topics of information searched when in remission by gender (A), type of disease (B) and age (C).

Regardless of the phase, women searched significantly more often for tips related to daily disease management, as compared to men (Figs 2–4). During active disease phases, patients aged less than 35 were significantly more concerned by information on therapies, (Fig 3), whereas patients aged over 50 were more concerned by general information on the disease. Finally, we observed that, at diagnosis, French-speakers searched significantly more on research (45.0% vs. 24.0%; p<0.001) and tips for daily disease management (33.8% vs. 18.2%; p = 0.005) as compared to German-speakers. French-speakers searched also more frequently on research during remission phases (65.3% vs. 50.6%; p = 0.014), as compared to German-speakers.

During the FG discussions, UC patients expressed more needs for information and less satisfaction with the information received, in comparison with CD patients. UC patients were willing to receive much more information about the disease in general, its chronicity, what was happening inside their body, and the fact that medication would be life-long. Such information was perceived as essential to patients especially at the time of diagnosis, and considered that it should be given by gastroenterologists. Patients expected to be given information about the course of the disease, and considered the physician to have the responsibility of helping them with acceptance of their condition. They also felt that they were not given sufficient information about medication side effects, treatments in general, influence of nutrition on their symptoms, and extra-intestinal manifestations. UC patients considered information about their condition to be frequently given in dribs and drabs, and wished to be provided with plain literature, instead of having to deal with medical jargon. Sources of information reported by UC patients were essentially gastroenterologists and other physicians, friends working in medical fields, internet, and sometimes online discussion forums. They expressed mistrust and fear in the information found on the internet. Furthermore, UC patients considered coordination between gastroenterologists and other specialists or general practitioners to be insufficient, and that this lack of information-sharing could lead to potential delays or diagnosis errors. They were particularly concerned about stressing issues, social pressure and stigmatization, especially in professional environments.

CD patients were globally more satisfied with the information received, although they reported a lack of information on several topics, e.g. about the risks of smoking, extra-intestinal manifestations, symptoms, and medication side effects. They reported a need for clear information in order to be able to talk about the disease with physicians. However, they also considered that being given too much information about the disease, especially at the time of diagnosis, could be perturbing and fearsome. Reported sources of information for CD patients were physicians, research, TV programs, and personal experience. CD patients were also interested in stress influence on their symptoms, impact of their disease on their daily family life, and heredity.

### Factors associated with information-seeking activity

We observed that women and German-speakers UC patients were at higher probability of searching for information at the time of first symptoms (OR = 1.78; p = 0.020 and OR = 1.68; p = 0.048 respectively). At diagnosis, those who searched significantly more for information were women, with CD (OR = 1.82; p = 0.003) or UC (OR = 1.56; p = 0.049), and French-speaking CD patients (OR = 1.61; p = 0.022). During active disease phases, CD patients having a penetrating disease behaviour (OR = 1.96; p = 0.005), student UC patients (OR = 9.70; p = 0.034), Table 4, or CD reporting a high level of perceived stress (OR = 2.47; p = 0.003), as shown in Table 5, were at higher risk of searching for information.

**Table 4:** Adjusted* odds ratios (95% confidence intervals) for searching information when disease is active or quiescent, stratified by diagnostic.

	Active CD		CD in remission		Active UC		UC in remission	
	N (%)	OR (95%CI)	N (%)	OR (95%CI)	N (%)	OR (95%CI)	N (%)	OR (95%CI)
**All**	**207 (50.9)**		**160 (39.3)**		**175 (54.7)**		**124 (38.6)**	
**Female gender**	116 (51.8)	1.12 (0.76–1.67)	88 (39.3)	1.04 (0.69–1.57)	93 (58.9)	1.39 (0.88–2.18)	54 (34.2)	0.70 (0.44–1.11)
**Age** ^$^	44.8 (13.6)	1.00 (0.98–1.01)	46.0 (15.1)	**1.01 (1.00–1.03)**	42.0 (12.5)	**0.97 (0.95–0.99)**	45.7 (13.0)	1.01 (0.99–1.02)
**French speakers**	75 (48.4)	0.88 (0.59–1.32)	67 (43.2)	1.35 (0.89–2.05)	59 (52.2)	0.85 (0.52–1.38)	51 (45.1)	**1.62 (1.00–2.63)**
**Disease duration**^$^	13.8 (9.7)	1.01 (0.99–1.04)	13.2 (9.8)	0.99 (0.97–1.01)	10.8 (9.3)	1.02 (0.99–1.05)	11.4 (9.9)	1.01 (0.98–1.03)
**Married**	93 (50.0)	0.78 (0.50–1.22)	77 (41.4)	0.90 (0.57–1.42)	97 (54.2)	1.01 (0.61–1.65)	74 (41.1)	1.20 (0.73–1.98)
**Family history of IBD**	26 (56.5)	1.30 (0.69–2.44)	20 (43.4)	1.22 (0.65–2.30)	20 (52.6)	0.82 (0.41–1.65)	15 (38.4)	1.03 (0.51–2.08)
**Education level**								
*None or compulsory*	27 (56.3)	1.00	19 (39.6)	1.00	17 (46.0)	1.00	11 (29.7)	1.00
*Upper 2nd education*	108 (46.0)	0.68 (0.36–1.28)	88 (37.5)	0.96 (0.50–1.84)	85 (52.2)	1.22 (0.58–2.57)	61 (37.2)	1.40 (0.63–3.08)
*Tertiary education*	64 (58.2)	1.14 (0.56–2.29)	47 (42.7)	1.21 (0.59–2.47)	69 (61.6)	1.83 (0.82–4.05)	50 (44.6)	1.89 (0.81–4.35)
**Working status**								
*Employed*	135 (52.9)	1.00	93 (36.5)	1.00	123 (53.7)	1.00	88 (38.3)	1.00
*In training*	7 (31.8)	0.54 (0.20–1.47)	12 (54.6)	**3.59 (1.38–9.35)**	12 (92.3)	**9.70 (1.19–78.9)**	2 (15.4)	0.29 (0.06–1.45)
*At home/unemployed*	28 (51.9)	0.86 (0.46–1.60)	25 (46.3)	1.29 (0.68–2.42)	20 (60.6)	1.23 (0.56–2.70)	14 (42.4)	1.33 (0.61–2.87)
*Retired*	26 (47.3)	0.65 (0.33–1.28)	23 (41.8)	0.72 (0.36–1.44)	14 (45.2)	1.10 (0.43–2.81)	15 (48.4)	1.25 (0.49–3.13)
**Current smoker**	79 (53.0)	1.14 (0.75–1.73)	55 (36.9)	0.89 (0.58–1.36)	22 (48.9)	0.69 (0.36–1.32)	21 (46.7)	1.50 (0.78–2.86)

^$^mean (SD)

*adjusted for age, gender, disease duration

**Table 5:** Adjusted* odds ratios (95% confidence intervals) for searching information when disease is active or quiescent, stratified by diagnostic.

	Active CD		CD in remission		Active UC		UC in remission	
	N (%)	OR (95%CI)	N (%)	OR (95%CI)	N (%)	OR (95%CI)	N (%)	OR (95%CI)
**All**	**207 (50.9)**		**160 (39.3)**		**175 (54.7)**		**124 (38.6)**	
**Depression**								
*None*	154 (48.7)	1.00	117 (37.0)	1.00	146 (54.1)	1.00	102 (37.8)	1.00
*Mild*	28 (60.9)	1.47 (0.77–2.80)	26 (56.5)	**2.28 (1.20–4.32)**	19 (63.3)	1.48 (0.66–3.29)	16 (53.3)	1.80 (0.83–3.89)
*Moderate to severe*	17 (56.7)	1.40 (0.65–3.01)	11 (36.7)	0.92 (0.42–2.04)	7 (53.9)	0.92 (0.29–2.93)	4 (28.6)	0.65 (0.19–2.17)
**Anxiety**								
*None*	118 (46.3)	1.00	95 (37.2)	1.00	120 (53.6)	1.00	86 (38.4)	1.00
*Mild*	40 (52.0)	1.26 (0.75–2.12)	37 (48.0)	1.55 (0.92–2.61)	32 (58.2)	1.22 (0.66–2.24)	23 (41.8)	1.17 (0.63–2.14)
*Moderate to severe*	41 (68.3)	**2.57 (1.39–4.73)**	22 (36.7)	1.04 (0.57–1.90)	20 (58.8)	1.08 (0.51–2.30)	13 (37.1)	0.98 (0.46–2.08)
**Perceived stress**								
*None to low*	82 (45.1)	1.00	65 (35.7)	1.00	69 (47.6)	1.00	51 (35.2)	1.00
*Low to average*	42 (53.9)	1.50 (0.87–2.58)	36 (46.1)	1.67 (0.96–2.89)	40 (58.0)	1.47 (0.81–2.66)	30 (43.5)	1.48 (0.81–2.68)
*Average to High*	32 (49.2)	1.23 (0.69–2.19)	27 (41.5)	1.33 (0.74–2.39)	32 (62.8)	1.86 (0.96–3.63)	17 (33.3)	0.93 (0.47–1.84)
*High*	45 (65.2)	**2.47 (1.36–4.49)**	28 (40.6)	1.40 (0.77–2.52)	31 (64.6)	1.82 (0.91–3.64)	24 (49.0)	**1.98 (1.01–3.88)**
**Positive social support (ESSI)**^$^	24.0 (5.1)	0.98 (0.94–1.02)	24.1 (4.8)	0.99 (0.95–1.04)	24.6 (5.0)	0.98 (0.94–1.02)	25.7 (5.1)	1.00 (0.95–1.04)
**Re-experiencing PTSDS**^$^	2.1 (2.8)	**1.14 (1.04–1.25)**	2.1 (2.7)	**1.09 (1.01–1.19)**	2.0 (2.2)	**1.21 (1.07–1.38)**	1.9 (2.1)	**1.12 (1.01–1.25)**
**Avoidance PTSDS**^$^	3.7 (4.1)	**1.11 (1.05–1.18)**	3.3 (3.9)	1.03 (0.98–1.09)	3.1 (3.3)	**1.14 (1.05–1.24)**	3.1 (3.3)	**1.08 (1.00–1.16)**
**Hyperarousal PTSDS**^$^	3.8 (3.3)	**1.13 (1.05–1.21)**	3.7 (3.4)	**1.09 (1.02–1.16)**	3.0 (2.5)	**1.13 (1.03–1.25)**	2.9 (2.7)	1.05 (0.96–1.14)
**SF-36 physical component**^$^	47.2 (43.3)	0.98 (0.96–1.00)	46.8 (10.3)	0.98 (0.96–1.00)	48.6 (9.3)	**0.96 (0.93–0.99)**	48.9 (9.1)	0.99 (0.96–1.01)
**SF-36 mental component**^$^	43.3 (11.9)	**0.96 (0.95–0.98)**	44.4 (11.1)	0.98 (0.97–1.00)	45.0 (10.6)	0.97 (0.95–1.00)	44.7 (11.3)	**0.97 (0.95–0.99)**
**IBDQ bowel sub-score**^$^	54.4 (10.2)	**0.95 (0.93–0.97)**	55.6 (10.1)	0.98 (0.96–1.00)	56.3 (10.0)	**0.96 (0.94–0.99)**	57.7 (10.7)	0.99 (0.97–1.02)
**IBDQ systemic sub-score**^$^	23.7 (6.2)	**0.95 (0.92–0.98)**	24.2 (6.2)	0.98 (0.95–1.02)	24.4 (5.7)	**0.92 (0.89–0.96)**	25.1 (6.1)	0.98 (0.95–1.02)
**IBDQ emotional sub-score**^$^	63.1 (12.8)	**0.97 (0.95–0.98)**	64.0 (12.4)	0.98 (0.97–1.00)	64.5 (11.5)	**0.96 (0.94–0.98)**	65.3 (11.7)	0.98 (0.96–1.00)
**IBDQ social sub-score**^$^	29.2 (6.8)	**0.95 (0.92–0.98)**	29.5 (6.5)	0.98 (0.94–1.01)	30.2 (6.4)	**0.93 (0.89–0.97)**	30.0 (6.8)	0.96 (0.92–1.00)

PTSDS = posttraumatic stress disorder symptoms

^$^mean (SD)

*adjusted for age, gender, disease duration

The risk of searching for information in UC with active disease (OR = 0.97; p = 0.002) decreased with increasing age. All patients with posttraumatic stress—regardless of the type of symptoms—were at higher risk of searching for information when disease was active. This risk decreased when quality of life increased. CD patients with a high level of perceived stress (OR = 2.47; p = 0.003) or moderately to severely anxious (OR = 2.57; p = 0.002) also tended to search more actively for information when their disease was active. In contrast, factors significantly associated with information-seeking during remission in CD patients were age (OR = 1.01; p = 0.002), being in training (3.59; p = 0.009), suffering from re-experiencing or hyperarousal posttraumatic stress symptoms (OR = 1.09; p = 0.008) or experiencing mild depression symptoms (OR = 2.28; 0.011) (Table 5). In UC, being French speaker, having a high level of perceived stress (OR = 1.98; p = 0.034), and experiencing avoidance or re-experiencing posttraumatic stress symptoms were associated with information-seeking behaviours in remission (Table 5).

## Discussion

This study aimed at assessing information-seeking activity, sources and topics of information among patients suffering from IBD in Switzerland. We found that almost half of the patients searched for information, regardless of the disease stage, but slightly less (39%) when disease was in remission. CD patients seemed to have been more satisfied with the information received than UC patients. Depending on disease phases, overall information needs varied from general information on the disease (at diagnosis) to drugs and therapies (during a flare), and research & development (in remission), and daily disease management. In general, women searched significantly more often for tips related to daily disease management, as compared to men. French-speakers patients were more active information seekers, as compared to German-speakers. Perceived stress levels and posttraumatic stress symptoms were associated with higher information-seeking activity; on the opposite having a better health-related quality of life tended to lower this activity.

Our results underline the need for more information among patients suffering from IBD, as described in previous studies [2–4,9,34]. Concerning satisfaction, we interestingly found the same proportions of patients dissatisfied with information (27%) at the time of first symptoms as Bernstein et al. shortly after diagnosis[4]. Aside from an objective lack of information that may exist during these specific periods, this might also be indicative of the emotional situation and uncertainty patients are leaving at that time that render them unable to accept or gain information. We also found that CD patients were less frequently fully satisfied, in general, as well as women who seemed to challenge information from physicians, but also internet and books or TV. Knowing that one of the main reasons of dissatisfaction was related to diagnostic delay, these observations are in line with previous observations showing that female gender was associated with diagnostic delay in IBD [35]. In comparison with the study of Bernstein et al [3,9], we did not assess who exactly diagnosed IBD for those patients, thus could not further discuss the relation between lack of information and medical specialty. Our study confirmed that gastroenterologists and medical professionals in general were the most consulted source of information for IBD patients [9,14]. Nurses were not frequently cited by patients as a source of information at diagnosis, which is probably linked to the fact that IBD nurses do not exist in Switzerland. IBD nurses could have been an important resource for gathering general information on the disease, especially at that time point. Internet was cited frequently in the survey, but less in the focus groups. At diagnostic, internet was a more frequent source of information for Swiss patients than Italian [7,17] or Canadian [9] patients. Books or TV were used by one quarter of the patients to gather information at the time of first symptoms or at diagnosis. Those media might be seen as a frequent way to complement information founded through other sources. Swiss patients association was consulted by a very limited number of patients, which was very surprising. This might be explained by the fact that patients enrolled in a cohort study might have different expectations as compared to those from associations. They probably search more frequently for factual information and are less prone or interested by exchanges of disease experiences.

Our study shows that information needs, as well as consulted sources of information, could vary depending on the course of the disease, confirming previous observations [36]. IBD patients wished to be given more information about long-term disease progression [9], drugs and treatments, diet [3,6,17] or complications [37,38], topics which were also cited in our study. We found that general information was more important to patients at the time of diagnosis, whereas information about therapies was more important in periods of flares. This was confirmed by the focus groups in which patients expressed a need for more information about what was happening inside their body at the time of diagnosis. Long-term disease progression, available treatment options and their potential side effects were then of more of interest once having experienced the disease for a longer time. Information needs evolved with disease duration and were persistent over years of experience of the disease. Indeed, we found that information about the disease was sought for mostly during relapse and when patients had lived with the disease long enough to realize they had not received enough information about their condition. The important needs for information expressed by participants in our focus groups could thus be justified by their long-standing disease experience. Focus groups emphasized that information should be provided to patients progressively and sparingly, along with their experience of the disease and treatments. In that regard, it appeared that the information given by gastroenterologists was insufficiently balanced with patients’ actual experience of the disease. Additionally, we found that age and gender of patients was associated to search for specific topics of information. Women and young patients were more frequently interested by information related to daily disease management, especially about nutrition and how to prevent new flares.

The impact of IBD on patients’ daily life has been explored by previous qualitative studies, showing that the disease interfered with patients’ social and family life [37,39] and was found to negatively impact on health-related QoL [40]. Our results show that patients with increasing scores of health-related QoL tended to search significantly less for information. In addition, according to our results, stress seemed to be the most important factor associated with a higher need to search for information. To date, no studies assessing the impact of information on stress was done, although stress was shown as a time lag factor associated with disease recurrence [41]. Stress was the most frequent psychological factor cited by patients as having had an influence on disease recurrence. Furthermore, they indicated that listening and help on how to cope with it is currently lacking.

We found that UC and CD patients may experience different worries and concerns. Other studies [34] have tried to show differences between UC and CD patients, but it seems that although some differences were found, these were not further explored in the literature. In our study, differences related to the needs and concerns appeared between UC and CD. Indeed, CD patients were much more satisfied than UC patients with the information they received on their disease, and also expressed fewer worries and concerns about their professional and social lives than UC patients. According to qualitative observations, UC patients appeared to be more focused on their social life and the difficulties in sharing their experience with others, whereas CD patients were more concerned about surgery, risks of smoking and heredity. Finally, we found interesting and never observed so far differences across linguistic regions. French-speakers seemed to be more active information seekers than German-speakers, although they are living within the same healthcare system. These might be related to cultural differences, but we found no other data to make a clear comparison or conclusion, although variations in medicine and culture, thus in information or expectations related have been explored since a long time [42]. Only one study showed important cross-cultural differences in specific concerns linked to IBD [43].

Among the strengths of our study was the opportunity to use data from a national clinical cohort of patients from different linguistic areas of Switzerland, being followed in various medical settings such as private practice, regional hospitals or university centres, to characterize patients who searched for information. The open-questions used for the survey had the strength of giving the patients space to express themselves freely, allowing deeper exploration of patients’ representations than surveys using close-ended questions. Combining quantitative and qualitative data with contents drawn from focus group discussions allowed even further comprehension of patients’ information needs, worries and concerns related to their chronic condition.

A limitation to our study might be linked with the generalisation of the results to the whole IBD population, knowing that some differences were noticed comparing responders and non-responders to the survey, even if those were in accordance with characteristics of non-responders found in one of our previous study [44]. Generalisation of results concerning the internet might also be limited for countries where internet access is restricted or limited, which will obviously affect the amount of information that patients could find by themselves. Another limitation related to the latter is the fact that participating in the cohort study itself reflects an interest for research and information about the disease, which may not be as important in a general population of IBD patients, for example this of patient associations. Ideally, this study should be conducted also through members of the Swiss patient association to get a broader and complete picture of overall information needs.

In conclusion, our results tended to indicate that information remains insufficient for IBD patients on many aspects of their disease. This study emphasizes that information needs can be different in various patient groups, at least according to gender, age and type of disease. Appropriate information and better understanding of the patients’ needs and concerns should be considered as potentially important components to improve patient-related outcomes, such as adherence to treatment, quality of life or stress coping.
